# Case Report: Exceptional Response to Poziotinib in Patient with Metastatic Non-Small Cell Lung Cancer With EGFR Exon 20 Insertion Mutation

**DOI:** 10.3389/fonc.2022.902967

**Published:** 2022-06-08

**Authors:** Arsela Prelaj, Achille Bottiglieri, Gajanan Bhat, Rocky Washington, Giuseppina Calareso, Gabriella Francesca Greco, Roberto Ferrara, Marta Brambilla, Alessandro De Toma, Mario Occhipinti, Sara Manglaviti, Alberto Soro, Monica Ganzinelli, Giuseppe Lo Russo, Claudia Proto

**Affiliations:** ^1^ Medical Oncology Department, Fondazione IRCCS Istituto Nazionale Tumori, Milan, Italy; ^2^ Department of Electronics, Information and Bioengineering, Politecnico di Milano, Milan, Italy; ^3^ Research and Development, Spectrum Pharmaceuticals, Irvine, CA, United States; ^4^ Department of Radiology, Fondazione IRCCS Istituto Nazionale dei Tumori, Milan, Italy; ^5^ Postgraduate School of Radiodiagnostics, Università degli Studi di Milano, Milan, Italy

**Keywords:** poziotinib, NSCLC, lung cancer, exon 20 insertion mutation, EGFR, exon 20 insertion (ex20ins), complete response (CR), target therapy

## Abstract

Among the several next-generation tyrosine kinase inhibitors (TKIs) tested against uncommon EFGR alterations, poziotinib has been demonstrated to be a powerful agent for metastatic non-small-cell lung cancer (mNSCLC) with aberrations in *HER2* exon 20, and FDA approval is being sought in the previously-treated population. Poziotinib has also shown activity in mNSCLC with aberrations in EGFR exon 20. Herein, we report the first published case of a patient affected by mNSCLC harbouring an *EGFR* exon 20 insertion (EGFRex20ins) mutation who achieved a complete response (CR) under treatment with poziotinib as part of the ZENITH20 trial. In January 2021, a former smoker 62-year-old female patient was diagnosed with relapse, after two surgeries and post-operative chemotherapy of mNSCLC, at liver and retroperitoneal nodes. Given the identification by Next Generation Sequencing (NGS) of EGFRex20ins mutation, she was enrolled in ZENITH20-cohort 5 trial, a phase 2 multicentre study aimed to assess the efficacy and safety of poziotinib in patients with EGFR or HER2 exon 20 insertion mutations. Poziotinib as first-line systemic therapy for metastatic disease was initiated at the end of January 2021 and administrated at the initial dosage of 8 mg orally twice daily (BID). The most common side effects from the beginning of the treatment included alopecia, macular skin rash, diarrhoea, xerostomia, and conjunctivitis. Due to these adverse events, poziotinib was discontinued during the first 3 months and then reduced to 6 mg orally BID in April 2021. After the dose de-escalation, the adverse events ameliorated, and the patient better tolerated the treatment without further interruption. Since the first reevaluation (after 4 weeks of therapy), the treatment with poziotinib resulted to be remarkably effective, with a partial response (PR) subsequently confirmed in May and July 2021. Then, in October 2021, a CT scan confirmed a CR, maintained with good tolerance at the last reevaluation in February 2022. Treatment is still ongoing at the same dosage. In this case, poziotinib has represented a successful and well-tolerated first-line treatment alternative to chemotherapy in this patient with EGFR exon 20 insertion mutated mNSCLC.

## Introduction

*EGFR* gene activating mutations are detected in approximately 10%-15% of Caucasian patients with metastatic non-small-cell lung cancer (mNSCLC). The most common mutations encompass exon 19 deletions and L858R exon 21, which respond convincingly to first, second, and third generation *EGFR* tyrosine kinase inhibitors (TKIs) (1).

Amongst the uncommon *EGFR* mutations, exon 20 insertions (EGFRex20ins) and duplications account for approximately 4%-10% of *EGFR* driver aberrations and are generally resistant to targeted therapy with TKIs due to inaccessibility of the binding site for this mutation (2). For these patients, the novel targeted therapies amivantimab-vmjw or mobocertinib have been approved only for use as subsequent therapy, and conventional platinum-based chemotherapy with or without bevacizumab remains the standard of care in first line therapy (1, 3).

Several ongoing trials are investigating the efficacy of next-generation TKIs against uncommon *EGFR* alterations and in particular EGFR ex20ins in first line treatment, including mobocertinib (4, 5), amivantamab with or without lazertinib (6, 7), and poziotinib (8). In particular, poziotinib is a powerful antagonist of the most common *EGFR* and *HER2* exon 20 insertion mutations (9, 10). In the phase 2 ZENITH20 trial (ClinicalTrials.gov Identifier: NCT03318939), manageable safety and clinically meaningful efficacy have been reported for patients with treatment-naïve NSCLC harbouring *EGFR* (8) and *HER2* exon 20 insertion mutations (9). To our knowledge, herein, we present the first published case of a patient affected by mNSCLC harbouring an EGFRex20ins mutation who achieved a complete response (CR) under treatment with poziotinib. This patient was enrolled in Cohort 5 of the ZENITH20 trial.

## Case Description

In 2017, a 58-year-old female patient who was a former smoker (25 pack/years) underwent right lobectomy and lymphadenectomy for a stage IB diagnosis of NSCLC (stage pT2aN0, according to the 7^th^ lung cancer TNM classification and staging system).

According to stage, adjuvant chemotherapy was indicated and proposed, but patient refused. Adequate and regular follow-up was initiated after surgery.

In January 2020, a single site cancer relapse at the right adrenal gland was detected by 18-FdG-PET and then removed by robotic surgery: the pulmonary origin was histologically confirmed, and the EGFR exon 20 p.D770_N771insSVD mutation was identified by Next Generation Sequencing (NGS) analysis. Due to the oligometastatic disease, four cycles of post-surgical chemotherapy with cisplatin and pemetrexed were administrated.

One year later, in January 2021, several new metastases in the liver and in retroperitoneal nodes were observed by CT scan. Due to the presence of EGFRex20ins mutation and the compliance with the other eligibility criteria, patient was enrolled in cohort 5 of the ZENITH20 trial. ZENITH20 is a multicentre, multicohort, open-label phase 2 study, aiming to evaluate the efficacy and safety of poziotinib in patients with mNSCLC. In particular, the Cohort 5 (ZENITH20-5) includes patients with mNSCLC harbouring *EGFR* or *HER2* exon 20 insertion mutations with or without prior treatment. Therapy with poziotinib was initiated at the end of January 2021 and administrated at 8 mg orally twice daily (BID) as a starting dose.

Since the beginning of the treatment in February 2021, alopecia, macular skin rash, diarrhoea, xerostomia, and conjunctivitis were reported as the most common drug-related (DR) adverse events.

Treatment course occurred as follows: a first dose interruption of approximately 10 days occurred 16 days after starting poziotinib and was due to unknown origin fever G3, likely DR. When she recovered (G0) after antibiotic-based therapy, patient re-started poziotinib at the original 8 mg BID dose. In March 2021, due to skin rash G3 and worsening conjunctivitis G2, poziotinib was stopped again for approximately 7 days. After topical treatments based on hydrocortisone 2.5% and doxicicline 100 mg once a day, the skin rash toxicity ameliorated from G3 to G1 and the conjunctivitis from G2 to G1. Then, poziotinib was re-initiated at the same dose (8 mg BID).

**Figure 1:**
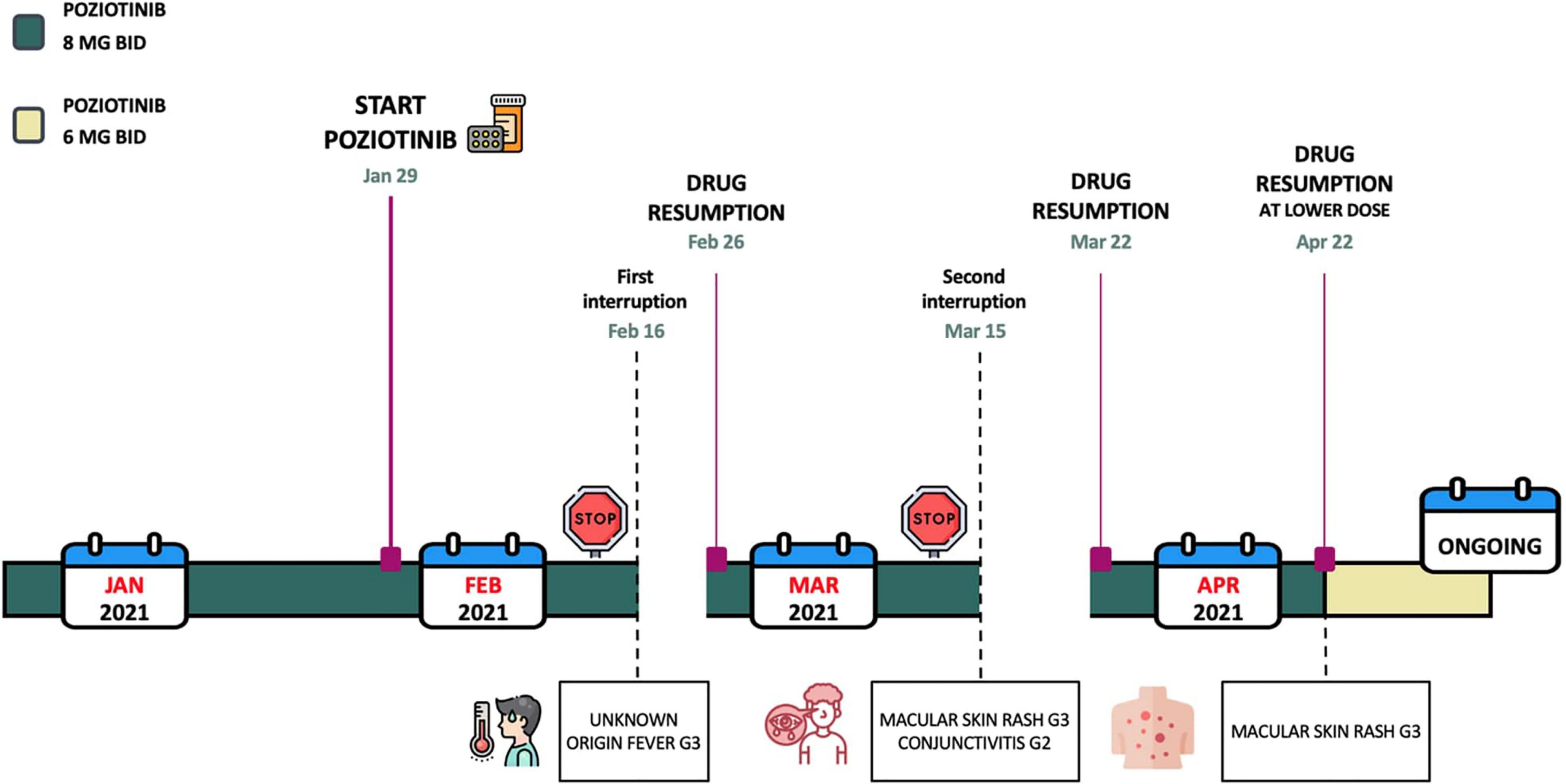
Here is reported the timeline of poziotinib administration and the related toxicities.

After 3 weeks, unfortunately patient presented with a recurrence of the skin rash G3. Therefore, as required by protocol, in April 2021 (98 days after first initiating poziotinib treatment) a dose reduction to 6 mg twice daily (12 mg in total/day) was performed. Consequently, the adverse events improved in severity and the patient better tolerated the treatment without further interruption. In **Figure 1**, the timeline of poziotinib administration with the resulting side effects is reported.

Since the first reevaluation (after 4 weeks), therapy with poziotinib resulted in being clinically effective, with the patient achieving partial response (PR) at the first radiological assessment.

**Figure 2:**
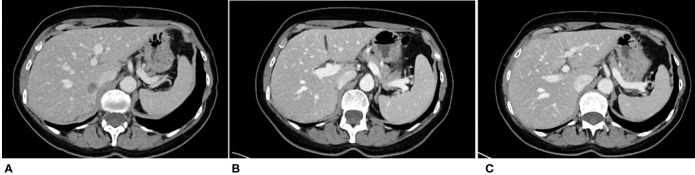
CT scans performed on January 2021 [**(A)**, baseline poziotinib] and subsequently on March 2021 **(B)** and October 2021 **(C)** demonstrated a PR **(B)** and a disappearance of the lesion **(C)**.

Later, in May and July 2021, a reduction in the overall tumour size of 79% and 84%, respectively, was recorded. Then, in October 2021, CT scan confirmed a CR (reduction on tumour size of 100%) on both the liver and the retroperitoneal nodes (**Figure 2**).

**Figure 3:**
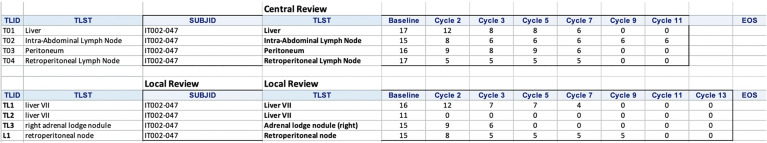
This figure demonstrated the exact lesions selected as target lesion for central and local radiological review.

**Figure 4:**
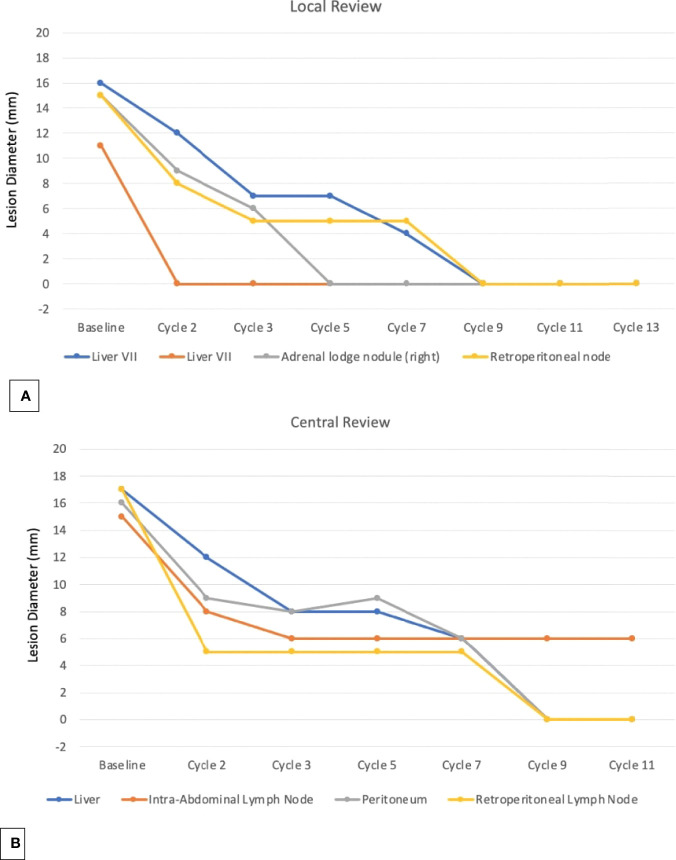
The graphics showed the local **(A)** and the central review **(B)** of the RECIS 1.1 criteria.

This radiographic evaluation was then confirmed by an independent review, except for a target lesion (an intra-abdominal lymph node), measuring less than 6 mm and considered as non-pathological by a radiological review opinion (**Figures 3**, **4**).

To further confirm the CR, a liquid biopsy with NGS Hotspot Panel was performed in January 2022, with negative result. This result supported the fact that no circulating minimal residual disease was present.

At the time of this report (April 2022) poziotinib therapy, at a dosage of 6 mg BID after 15 months is still ongoing. The CR has been maintained with good tolerance and toxicity not exceeding G1 (last CT scan was performed on April 2022).

## Discussion

Despite the treatment progress for the most common EGFR alterations in mNSCLC, EGFRex20ins mutations still represent an unmet clinical need, especially in the first line setting. The few novel targeted agents (amivantimab-vmjw, mobocertinib) have been approved only for subsequent therapy and a common well-established therapeutical algorithm is lacking.

Mobocertinib, an oral and irreversible TKI, selectively designed against EGFRex20ins mutations, was evaluated in the phase 1/2 open-label nonrandomized EXCLAIM trial, with interesting results: in the platinum-pretreated patients cohort, with a starting dose of 160 mg once a day, an overall response rate (ORR) of 28% (95% CI, 20%-37%) was reported by the independent review committee (IRC), compared to 35% (95% CI, 26%-45%) observed by investigators. The confirmed disease control rate by IRC assessment was 78% (95% CI, 69%-85%). At data cut-off, median IRC-assessed PFS was of 7.3 months (95% CI, 5.5-9.2), in line with how measured by investigators (7.3 months - 95% CI, 5.6-8.8). Most common drug-related (DR) side effects included diarrhoea, skin rash, and paronychia; in detail, 21% of patients suffered from diarrhoea G3 or worse (4).

However, the role of mobocertinib in the first line setting is still to be confirmed, and is currently under investigation by the phase 3, randomized EXCLAIM-2 trial (ClinicalTrials.gov Identifier: NCT04129502), aiming to compare its efficacy to platinum-based chemotherapy in treatment naïve patients with mNSCLC harbouring EGFRex20ins mutations (5).

While waiting for the results of EXCLAIM-2, in September 2021 the Food and Drug Administration (FDA) granted accelerated approval to mobocertinib as a subsequent treatment option in patients affected by mNSCLC harbouring EGFRex20ins mutations, after having progressed on or after platinum-based chemotherapy (11). This choice has not yet been followed by the European Medicine Agency (EMA), though mobocertinib was recently approved in the UK in March 2022.

On the other hand, FDA and EMA approved in January 2022 amivantamab, an EGFR-MET bispecific antibody with immune cell-directing activity, as a subsequent therapy in EGFRex20ins mutated mNSCLC after platinum-based chemotherapy failure (12). Safety and efficacy of amivantamab is under evaluation in different cohorts in the phase 1, open-label, dose-escalation, and dose-expansion CHRYSALIS trial. Unlike mobocertinib, amivantamab is administrated intravenously, once weekly for the first 4 weeks and then once every 2 weeks starting at week 5. In the post-platinum EGFRex20ins mutated population, an ORR of 40% (95% CI, 29% - 51%) was reported, with a median duration of response of 11.1 months (95% CI, 6.9 - not reached). The median PFS was 8.3 months (95% CI, 6.5 - 10.9). The toxicity profile was considered quite manageable: skin rash, infusion-related reactions, and paronychia were reported as the most common DR adverse events; nonetheless, hypokaliemia, skin rash, pulmonary embolism, diarrhoea, and neutropenia were described as occasional G3 side effects or worse (6).

In the first line setting, amivantamab is currently under investigation in association with lazertinib, a potent, brain-penetrant, 3rd-generation EGFR TKI, capable of targeting both EGFR and resistance T790M mutations: indeed, in the ongoing phase 3, multicentre, randomized MARIPOSA trial (ClinicalTrials.gov Identifier: NCT04487080), their combination efficacy will be compared to Osimertinib as the standard of care treatment for treatment naïve patients with NSCLC caused by mutations in the EGFR (7).

In this lively and still evolving treatment landscape, the oral pan-ErbB inhibitor poziotinib is being tested in both treatment naïve and previously treated patients diagnosed with EGFR or HER2 exon 20 insertion mutations.

Recently, the first results from Cohort 2 in patients with HER2 exon 20 mutations have been published: an objective response rate of 27.8% (95% CI, 18.9% - 38.2%) was described, while the disease control rate resulted to be 70.0% (95% CI, 59.4% - 79.2%). Median progression-free survival was 5.5 months (95% CI, 3.9 - 5.8); interestingly, clinical benefit was seen regardless of lines and types of prior therapy, presence of central nervous system metastasis, and types of HER2 mutations (9)

On the contrary, no data about the EGFRex20ins mutated cohorts of ZENITH20 has been published yet in a peer-reviewed journal. In the results of the Italian Expanded Access Program (EAP) reported by Prelaj et al. in 2021, the ORR in patients affected by EGFRex20ins mutations was 23%, with a median PFS of 5.6 months (95% CI: 3.6 - 6.7) (10); these results seem to be quite satisfactory considering the fact that these patients were heavily pre-treated and were quite similar to real life patients. Despite at first glance the efficacy of poziotinib seems inferior to amivantamab and mobocertinib, the worst general conditions of patients in the EAP and the high rate of interruption or discontinuation of the treatment, due to the burden of DR toxicities, might have affected the results. Moreover, these first EAP patients were all treated with the QD schedule (16 mg/day) meaning that drug-discontinuation was likely more frequent with the previous regimen.

In fact, skin rash, diarrhoea, and stomatitis were observed as the most common adverse events in both studies, leading to 76.7% of patients in cohort 2 of the ZENITH20 trial requiring a dose reduction (9). We have reported the same kind of concern in our clinical case, describing better compliance to the treatment, without any further interruption, once the dosage was diminished to 6 mg twice a day while still maintaining the tumour suppression in the responders.

Soon, it will be interesting to evaluate the outcomes of ZENITH20 trial Cohort 5 and whether, with an appropriate dosage adjustment and greater adherence to therapy, the results of ORR and PFS will be comparable with those of amivantamab and mobocertinib. As additional data becomes available for analysis, there will be an opportunity to better understand poziotinib efficacy in patients with EGFRex20ins mutations.

## Conclusion

EGFRex20ins mutations continue to represent an urgent unmet clinical need, especially in the front line setting. In fact, despite new molecules recently approved as subsequent targeted therapies, a consolidated and reliable therapeutic algorithm is still to be confirmed and chemotherapy remains the first line regimen.

This remaining lack of efficacious targeted treatment options and poor prognosis of newly diagnosed patients affected by mNSCLC harbouring EGFRex20ins justifies the endeavour to develop novel tailored drugs and maybe start to think on sequence for these patients looking at their short PFS compared to classical EGFR mutations.

In our case, we reported that in one patient with treatment naïve mNSCLC harbouring an EGFRex20ins mutation, poziotinib has represented a successful and well tolerated alternative compared to chemotherapy achieving a sustained complete response.

## Data Availability Statement

The datasets for this article are not publicly available due to concerns regarding participant/patient anonymity. Requests to access the datasets should be directed to the corresponding author.

## Ethics Statement

The studies involving human participants were reviewed and approved by Ethics Committee of Fondazione IRCCS Istituto Nazionale dei Tumori of Milan, Italy. The patients/participants provided their written informed consent to participate in this study.

## Author Contributions

AP and CP: investigation, methodology, supervision, writing—original draft, writing—review and editing. AB: writing—original draft, writing—review and editing. GB and RW: methodology, supervision, writing—original draft, writing—review and editing. GC, GG, and AS: investigation, supervision, writing—review and editing. RF: investigation, methodology, writing—review and editing. MB, ADT, MO, SM, and MG: investigation, writing—review and editing. GLR: investigation, methodology, supervision, writing—review and editing. All authors contributed to the article and approved the submitted version.

## Conflict of Interest

AP declares personal fees from Roche, AstraZeneca and BMS outside the submitted work. GL declares personal fees from BMS, MSD and Astra Zeneca outside the submitted work. CP declares personal fees from BMS and MSD, outside the submitted work. GB declares employment and stock ownership at Spectrum Pharmaceuticals, Inc. RW was employed by Spectrum Pharmaceuticals.

The remaining authors declare that the research was conducted in the absence of any commercial or financial relationships that could be construed as a potential conflict of interest.

## Publisher’s Note

All claims expressed in this article are solely those of the authors and do not necessarily represent those of their affiliated organizations, or those of the publisher, the editors and the reviewers. Any product that may be evaluated in this article, or claim that may be made by its manufacturer, is not guaranteed or endorsed by the publisher.
